# Output-orientated policy engagement: a model for advancing the use of epidemiological evidence in health policy

**DOI:** 10.1186/s12961-022-00955-7

**Published:** 2023-01-16

**Authors:** Emily Banks, Abby Haynes, Ray Lovett, Uday Narayan Yadav, Jason Agostino

**Affiliations:** 1 National Centre for Epidemiology and Population Health, The Australian National University, Canberra, ACT Australia; 2 Institute for Musculoskeletal Health, The University of Sydney and Sydney Local Health District, Sydney, NSW Australia; 3 School of Public Health, Faculty of Medicine and Health, The University of Sydney, Sydney, NSW Australia; 4 School of Medicine and Psychology, ANU, Canberra, ACT Australia; 5 National Aboriginal Community Controlled Health Organisation, Canberra, ACT Australia

**Keywords:** Epidemiology, Health policy, Research translation, Output-orientated policy engagement, Research impact

## Abstract

**Background:**

Use of epidemiological research in policy and practice is suboptimal, contributing to significant preventable morbidity and mortality. Barriers to the use of research evidence in policy include lack of research–policy engagement, lack of policy-relevant research, differences in policymaker and researcher practice norms, time constraints, difficulties in coordination, and divergent languages and reward systems.

**Approach and outcomes:**

In order to increase policy-relevant research and research uptake, we developed the output-orientated policy engagement (OOPE) model, in Australia. It integrates a foundational approach to engagement with cycles of specific activity focused around selected research outputs. Foundational elements include measures to increase recognition and valuing of policymaker expertise, emphasis on policy uptake, policy awareness of the research group’s work, regular policy engagement and policy-relevant capacity-building. Specific activities include (i) identification of an “output”—usually at draft stage—and program of work which are likely to be of interest to policymakers; (ii) initial engagement focusing on sharing “preview” evidence from this output, with an invitation to provide input into this and to advise on the broader program of work; and (iii) if there is sufficient interest, formation of a researcher–policy-maker partnership to shape and release the output, as well as inform the program of work. This cycle is repeated as the relationship continues and is deepened. As well as supporting policy-informed evidence generation and research-aware policymakers, the output-orientated model has been found to be beneficial in fostering the following: a pragmatic starting place for researchers, in often large and complex policy environments; purposeful and specific engagement, encouraging shared expectations; non-transactional engagement around common evidence needs, whereby researchers are not meeting with policymakers with the expectation of receiving funding; built-in translation; time and resource efficiency; relationship-building; mutual learning; policy-invested researchers and research-invested policy-makers; and tangible policy impacts. A case study outlines how the output-orientated approach supported researcher–policymaker collaboration to generate new evidence regarding Aboriginal and Torres Strait Islander cardiovascular disease risk and to apply this to national guidelines.

**Conclusion:**

Output-orientated policy engagement provides a potentially useful pragmatic model to catalyse and support partnerships between researchers and policymakers, to increase the policy-relevance and application of epidemiological evidence.

## Background

Public health policy is the primary mechanism through which epidemiological research achieves its effects, resulting in profound impacts on our daily lives [1]. Globally, the use of epidemiological research in policy and practice is suboptimal, contributing to significant preventable morbidity and mortality [2–4]. Most epidemiologists believe there is a moral imperative to increase the policy and practice impact of their work [4], which is augmented by the increasing need to demonstrate social and economic benefits of taxpayer-funded research [5, 6].

The literature on policymakers’ and practitioners’ use of research describes a range of barriers to policy impact. Inaccessibly written, inconclusive research findings and incompatible time frames are part of the problem, but much published research is simply unusable by stakeholders due to its poor policy and practice relevance and contextualization [7–10]. Some policymakers distrust the applicability of epidemiological data [11, 12].

Tackling such barriers requires more than improved reporting and dissemination of research. Epidemiologists must find ways to address the real-world needs of policymakers and practitioners [13]; focusing less on supply and more on demand [14], tackling current policy questions and grappling with the complex systems and “wicked” problems that are central to policymaking [15, 16]]. To this end, research–policy partnerships and other forms of cross-sector interaction are well established as the most productive means for advancing evidence-informed policy [13, 17–22]. These interactive models highlight the centrality of relationship-building, mutual learning and optimizing complementary cross-sector expertise [23, 24].

Interactive modes of work, however, have their own challenges. They are time-intensive and difficult to coordinate, and must contend with the divergent language, reward systems and practice norms of policymakers and researchers [25–29]. Furthermore, it can be hard for all parties to identify entrées for relationship development and to establish mutually beneficial platforms for collaboration. While such challenges are widely bemoaned, there are few practical proposals for addressing them. Therefore, there is a need for pragmatic models that can help to bridge the knowledge gaps, trust and engagement required to develop policy-relevant evidence that is robust and addresses the needs of local decision-makers.

In this paper, we present such a pragmatic model: output-orientated policy engagement (OOPE). This model of research–policy and practice engagement and partnership has been developed and applied over the past decade, with demonstrated success. Its development followed multiple unsuccessful attempts to engage with policymakers. In this paper, we describe the OOPE process, illustrate its application with a case study, reflect on its benefits and limitations, and consider the potential lessons for other research groups.

## OOPE model approach

OOPE is an invitation to policymakers, practitioners and other end-users to shape research outputs that our team has carriage of. It was devised by public health researchers over many years of working in multidisciplinary teams, in partnership with policymakers, practitioners and community. It brings to bear our motivation to improve health outcomes through better research, policy and practice, and our expertise and experience in epidemiology, implementation science, knowledge exchange and mobilization, Indigenous health and research methodologies, general practice and chronic disease.

**Fig. 1:**
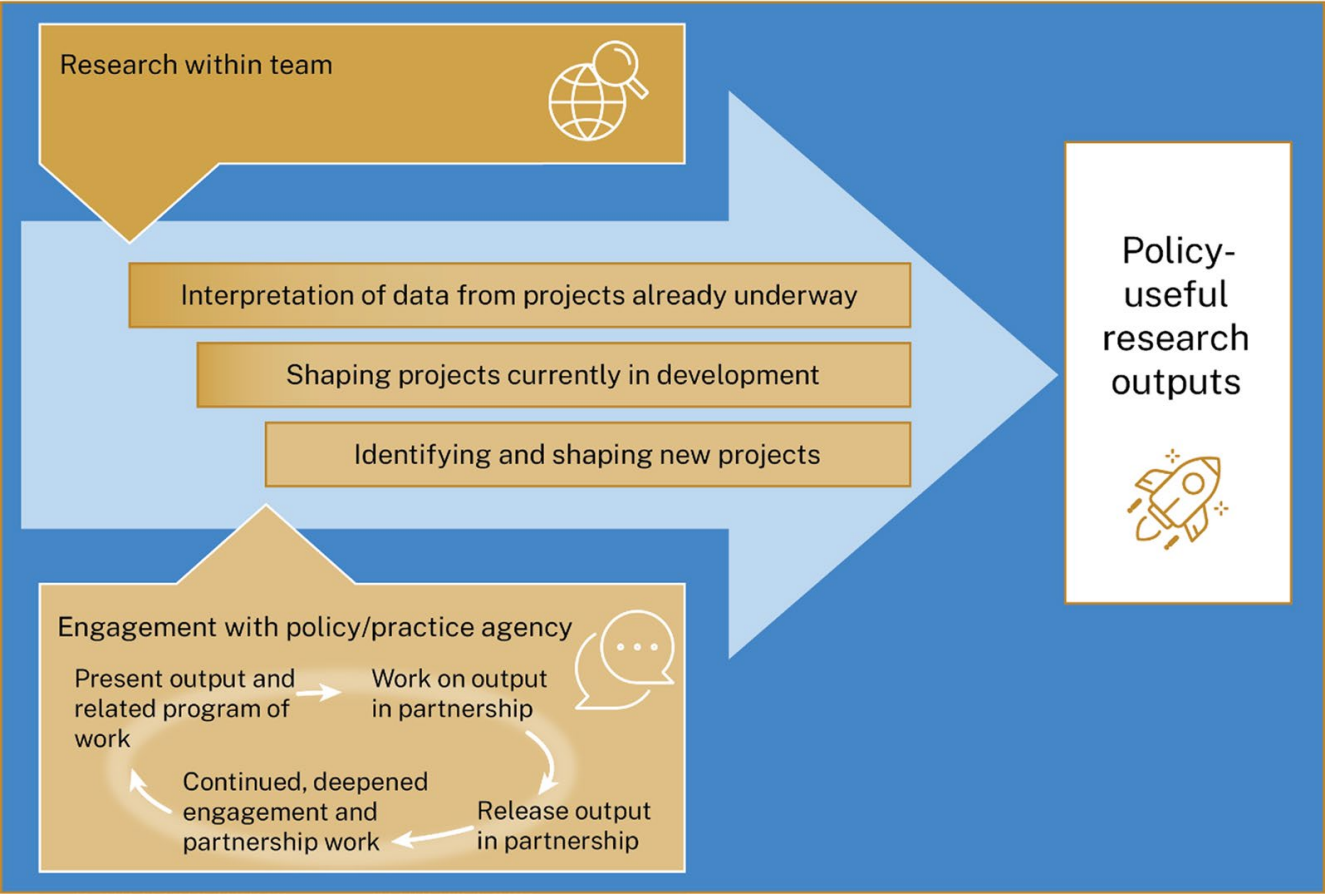
Output-orientated policy engagement process overview

OOPE integrates a foundational approach to engagement with cycles of specific activity focused around selected research outputs. *This cyclical approach allows us to act flexibly without losing the “active ingredients” that we believe make it work* (Fig. 1).

The foundational approach includes the following:

- culture-focused leadership and capacity-building to ensure that research group members recognized and value the expertise and input of policymakers, practitioners, community groups and other end-users;
- shifting the balance within research projects to increase the emphasis on policy uptake, including use of written outputs and presentations focused on policymakers’ needs, and policy-orientated dissemination and translation strategies;
- dedicating resources to generating awareness of the research group’s work with policy/practitioner/community groups, through multiple channels (e.g. media, conferences, strategic communication with policymakers and peak nongovernmental organizations);
- regular engagement with and work for policy and practice agencies (e.g. via meetings, conferences, provision of advice, responses to submissions and membership of government and nongovernmental committees);
- employment of research staff with skills in policymaking and clinical and public health practice, including engagement and implementation methods and capacity-building in these methods, such as the development of relevant “soft skills” and traits, including open listening, humility and resilience;
- involvement of policymakers, practitioners and community members as co-investigators throughout the research process with appropriate training and capacity-building.

The specific activities are described in the following:

### Identifying an “output” and program of work which is likely to be of shared interest

We start by identifying a specific output—usually the early results for a research paper or report—that we consider likely to be of interest to a particular policy audience. In general, this output would be at draft stage with early results available for discussion and would be part of a broader program of work, thereby proving scope to build on this research collaboratively.

### Initial engagement

Using initial contacts made at committee meetings, conferences and from other networks, we contact an individual within the relevant policy section and offer to share “preview” initial findings of the output, prior to publication, and to obtain their views on how to ensure its policy relevance. We ask the agency to invite anyone who might be interested and often suggest an “all-staff” presentation to inform on the background issues and research elements that are less confidential, and a more confidential higher level policy roundtable—for more senior staff who may want to input into the work. The presentations focus on the specific research product, providing information on its background—including summarizing the evidence to date—findings and implications, and asking attendees how its outputs can be maximized for policy use. Deliberations include thinking strategically about stakeholders, service users and end audiences for the research. This allows a unique space for interested policymakers and personnel to add their insights on how a specific research output could contribute to policy and practice.

Crucially, in this engagement phase we are offering something of potential value with no strings attached. We build this potential value into our work by meeting regularly with policymakers and by horizon scanning—both of which support keeping abreast of developments across policy, practice and research to identify where potential contributions can be made and striving to ensure our work addresses relevant research questions and produces applicable findings. This has become more sophisticated as we have built deeper relationships and learnt from our peers in policy and practice.

### Considering the program of work

We also use this opportunity to talk about related work within the same program but at different stages of development. The aim is to generate critical thinking about the focus of the research and its interpretation, to gather information about how the overall program of work could be shaped to meet policy needs. A secondary aim is to identify if/how the agency might be involved during the processes of idea inception, conducting the research and writing it up.

### Forming and furthering partnership work

In most cases, the policy agency accepts our invitation for ongoing input and so participates in shaping our work. This process is iterative and responsive to local conditions, generally involving several interactive presentations and at least one policy roundtable on key aspects of the research. Policy partners determine the scope of their involvement and contribution. This usually occurs at two levels: high-level strategic discussions with senior policymakers via periodic meetings, and a more intense “hands-on” collaboration between a small group of policymakers and researchers.

### Releasing the research output and positioning for implementation

In the final phase, we work in partnership to shape the form and release of the output and implementation of findings. We ensure that policy partners are well informed about publication timelines and have the opportunity to comment on and respond to the release of all outputs. Depending on their depth of engagement, they may opt for a coalition approach to release, participating in media opportunities and liaison with stakeholders.

### Continued engagement

As part of the cyclical approach, the research team identifies the next output from our program of work to focus on, drawing on policy interactions to prioritize and begins the next cycle of engagement, which is often at a deeper level as relationships and understanding have progressed. This next cycle often overlaps to some extent with a previous or subsequent cycle.

OOPE is very much a team approach: no individual researcher has the knowledge or time to manage it alone. Although led by a senior staff member, three or four team members attend every meeting, and a wide range of skills and specializations are tapped, according to the direction of the research. For presentations, we ask ourselves “who can speak to this” and “who should speak to it”, and strive to include those voices in a compelling narrative [28]. For example, clinicians lead on clinical issues, and community members speak to issues that affect them. Thus, we form connections with policymakers as scientists, as clinicians and as members of the communities that we seek to serve.

## Case study: ensuring early detection and management of cardiovascular disease risk in Aboriginal and Torres Strait Islander peoples

Cardiovascular disease (CVD) is the leading cause of death for Aboriginal and Torres Strait Islander people and a major cause of health inequity. This case study draws on work in partnership with Aboriginal and Torres Strait Islander health leaders and engagement of stakeholders from 2015 to 2021 for improving guidelines on CVD risk assessment in Aboriginal and Torres Strait Islander adults. The chronology of the case study is outlined in Table 1, along with how progress maps broadly against the OOPE approach, noting its nonlinear and iterative nature.

**Table 1:** Case study: Aboriginal and Torres Strait Islander CVD guideline development and alignment to ensure early detection and management of CVD risk

Timeline dates	Activity	OOPE focus
2003–present	Team- and system-based work on leadership and capacity-building, emphasis on policy needs, generating awareness of work, engagement with key stakeholders in CVD, population health and Aboriginal and Torres Strait Islander health, building research partnerships and producing policy-relevant research	Foundational work
2015–2016	Identification of absolute CVD risk assessment and management in the general population as an output of likely shared policy interest, within a broader programme of work Engagement with stakeholders on CVD risk assessment and management in the general population, including Department of Health, Heart Foundation, Australian Bureau of Statistics Input into general population paper and identification of Aboriginal and Torres Strait Islander absolute CVD risk as an area of interest for future work in the programme	Initial engagement, considering the broader program of work, forming and furthering partnership work
May 2016	Publication of paper on CVD risk assessment and management in the general population [2]	Release of research output and positioning for implementation
2016	Australian National University CVD program Aboriginal Reference Group identifies early CVD in Aboriginal and Torres Strait Islander peoples, prior to the age when assessment is recommended nationally, as a key concern	Forming and furthering partnership work and continued engagement
2016	Analyses of Australian Bureau of Statistics data on Aboriginal and Torres Strait Islander CVD risk, identifying early onset of risk, with results fed back to reference group. Commencement of drafting of paper	
June 2016	All-staff forum and roundtable 1: all-staff presentation and senior policy roundtable on early results on Aboriginal and Torres Strait Islander CVD risk at the Australian Government Department of Health	
June 2017	Work commissioned on risk assessment of CVD for Aboriginal and Torres Strait Islander population by the Department of Health; meetings every 3 weeks with staff from the Indigenous Health Division, Department of Health, from this time until June 2020, as part of this work	
November 2017	All-staff forum and roundtable 2: All-staff presentation and senior policy roundtable on implementation gaps in CVD prevention and proposed policy levers to improve uptake of absolute CVD risk at the Australian Government Department of Health. Roundtable focused on possible enhancements to Aboriginal and Torres Strait Islander health checks to improve CVD prevention	
December 2017	Submission of report to Australian Department of Health reviewing evidence for the alignment of guidelines on Aboriginal and Torres Strait Islander CVD risk	
February 2018	Stakeholder workshop on guideline alignment for Aboriginal and Torres Strait Islander CVD risk, including presentation of findings on absolute CVD risk in this population	
March 2018	Submission of report to the Department of Health outlining a strategy to support revision and alignment of guidelines for Aboriginal and Torres Strait Islander CVD assessment and management	Release of research output and positioning for implementation
April 2018	Findings on CVD risk in Aboriginal and Torres Strait Islander adults and Aboriginal and Torres Strait Islander health checks presented to the General Practice and Primary Care Clinical Committee Assessments Working Group of the Medicare Benefits Schedule Review	Continued engagement
June 2018	Australian National University CVD Aboriginal Reference Group supports lowering the age for commencing CVD risk assessment to 18 years and recommends communication strategies to promote community awareness of CVD risk assessment	Partnership work and continued engagement
June 2018	Workshop of representatives of organizations responsible for Aboriginal and Torres Strait Islander CVD guideline development: Australian Chronic Disease Prevention Alliance, Royal Australian College of General Practitioners (RACGP) and Central Australian Rural Practitioners Association. The group agreed on the need for systematic universal clinical CVD risk assessment from age 18 and to work together on a consensus statement	
June 2018	Paper on CVD risk in Aboriginal and Torres Strait Islander adults published online and launched by Minister Wyatt at Parliament House	Release of research output and positioning for implementation
July 2018	Findings on CVD risk in Aboriginal and Torres Strait Islander adults and Aboriginal and Torres Strait Islander health checks presented to Indigenous Reference Group of the Medicare Benefits Schedule Review	Continued engagement
June 2018–July 2019	Additional analysis on the prevalence of vascular risk factors in Aboriginal and Torres Strait Islander adults aged 18–29 was completed using data from the Australian Bureau of Statistics 2012–2013 Australian Aboriginal and Torres Strait Islander Health Survey. These results were presented to the Aboriginal Reference Group and Guidelines Working Group to guide discussions on extending a CVD risk assessment and management to adults younger than 30 [30] Drafting of consensus statement to lower age at CVD risk assessment in Aboriginal and Torres Strait Islander adults to 18 [31], with Aboriginal and Torres Strait Islander community representatives and leaders in CVD research as partners and authors, including representatives from guideline development groups: Heart Foundation, RACGP, National Aboriginal Community Controlled Health Organisation and the Central Australian Rural Practitioners Association Endorsement of consensus statement by the Heart Foundation Clinical Committee and Heart Health Committee, Chair of the RACGP Quality Care Committee and representatives from the Stroke Foundation, Kidney Health Australia and Diabetes Australia	Furthering partnership work, continued engagement and positioning for implementation
May 2019	Participation and presentation at roundtable held by RACGP on enhancements to Aboriginal and Torres Strait Islander health checks	Partnership work and continued engagement
August 2019	Consensus statement updating guidelines on CVD risk assessment in Aboriginal and Torres Strait Islander adults submitted to peer-reviewed journal	Release of research output and positioning for implementation
December 2019	All-staff forum and roundtable 3: all-staff presentation and senior policy roundtable on findings from review of Aboriginal and Torres Strait Islander health assessments and chronic disease prevention	Continued engagement
March 2020	Consensus statement updating guidelines on CVD risk assessment in Aboriginal and Torres Strait Islander adults published online [31]. This is the first time all relevant guidelines have been aligned for Aboriginal and Torres Strait Islander peoples and that age ranges for assessment have been based on representative empirical evidence	Release of research output and positioning for implementation
November 2020	National Aboriginal Community Controlled Health Organisation and RACGP publish Aboriginal and Torres Strait Islander health check templates that incorporate many of the key recommendations from the Australian National University review [34]	Release of research output, incorporating our research findings
February 2021	Paper on sociodemographic factors and CVD risk factors among Aboriginal and Torres Strait Islander peoples having health checks published [32]	Release of research output and positioning for implementation

**Fig. 2:**
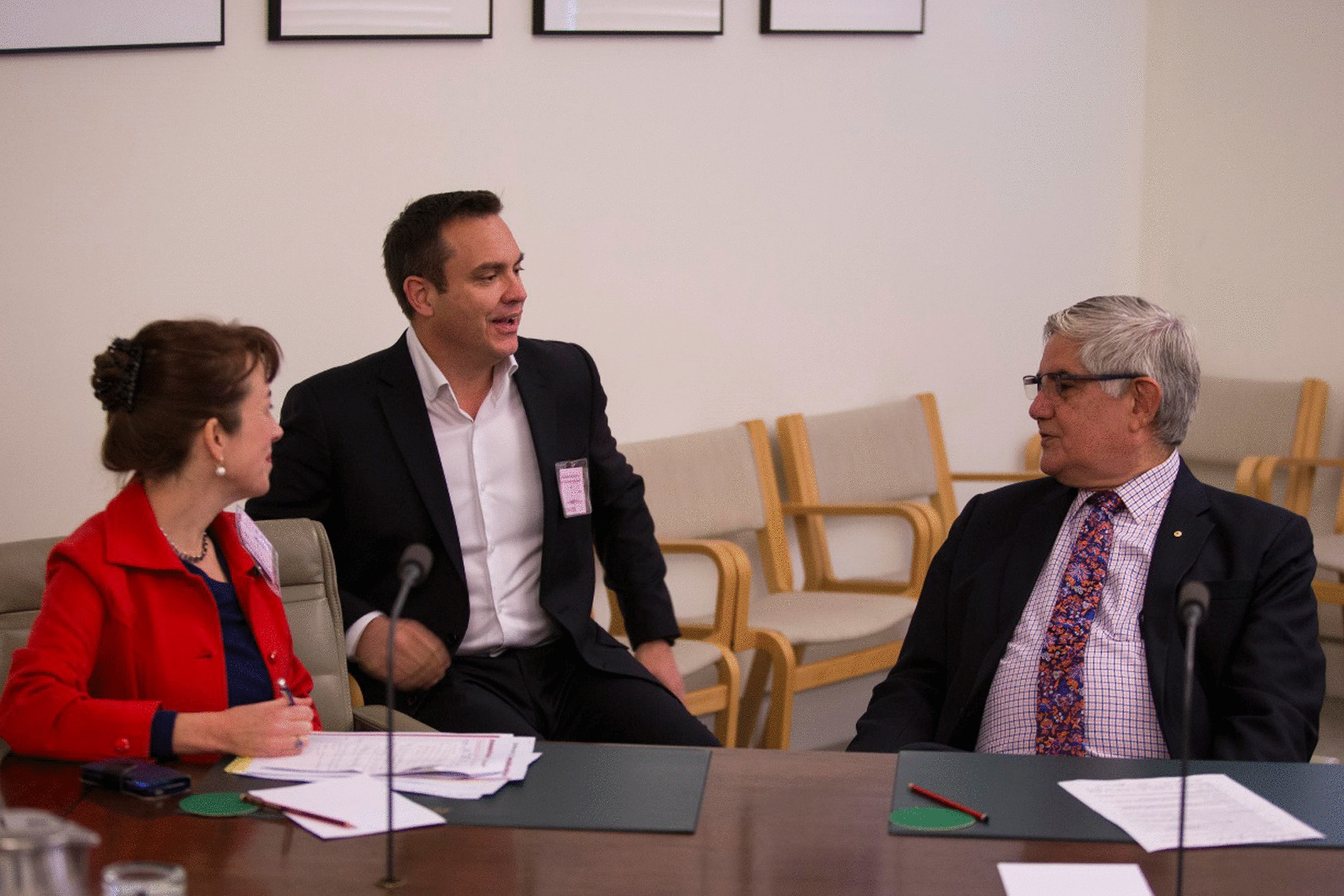
Behind the scenes at the launch of the paper on absolute cardiovascular risk in Aboriginal and Torres Strait Islander peoples, Parliament House, Australia, June 2018 (left to right: Professor Emily Banks, Professor Ray Lovett and the Hon Ken Wyatt, the then Minister for Indigenous Health. Photo credit: Jamie Kidston/The Australian National University)

Against a background of long-term and continuing foundational work to foster policy-relevant work and increase its impact, initial OOPE focused on collaborative work on quantifying the CVD risk profile of the general Australian population, with the Australian Bureau of Statistics and the National Heart Foundation of Australia [2]. This work was already underway, based on previous engagement, and work on CVD risk in Aboriginal and Torres Strait Islander peoples was identified with policymakers as an area of future work. Our Aboriginal Reference Group raised the issue that national guidelines for Aboriginal and Torres Strait Islander peoples start CVD risk assessment at age 35 and that CVD events in the community occur before this age. Aboriginal and Torres Strait Islander research leaders noted that this cutoff was based on consensus rather than evidence. Analyses of relevant data were undertaken, demonstrating that CVD risk in Aboriginal and Torres Strait Islander peoples is appreciable from age 18 onwards. This was presented to the Australian Department of Health, with the need for guideline alignment and implementation work raised as the next stage. This research was published [30] and launched by the then Minister for Indigenous Health, the Hon Ken Wyatt (Fig. 2). A workshop was also held. Our team was commissioned to work on a strategy to align guidelines which was subsequently implemented, with the outcome that a consensus statement updating national guidelines to start CVD risk assessment at age 18 in Aboriginal and Torres Strait Islander peoples was published in 2020 [31].

This first round of engagement with policy-makers on CVD risk assessment led to further collaboration on Aboriginal and Torres Strait Islander health checks. Health checks were identified as a focus of government policy and a tool for implementing earlier screening and improving CVD risk assessment and management. Repeating the OOPE cycle with research on health checks, we worked with the Department of Health and the Royal Australian College of General Practitioners (RACGP) to improve health check templates and guidance [32, 33].

## Benefits and limitations

From our experience of using this approach over the past decade, we have identified 10 key benefits:

### A place to start

Policy agencies are large and complex, and it is often unclear how best to engage. Focusing on a specific output using a straightforward, pragmatic approach allows researchers to start the process effectively, with evidence that is likely to be of benefit to policymakers.

### Policy-useful research outputs

Poor consideration of utility is a key factor in research wastage [34], and an enduring problem for policymakers who are often faced with a mismatch between their most pressing questions and academic research findings [35]. OOPE enables us to identify local policy priorities, hone our methods and outputs so that they are fit for purpose, and interpret findings in relation to the complex landscape that they will need to impact. 

### Purposeful engagement

OOPE provides a tightly focused forum for researchers and policymakers to start a productive conversation, which also has a clear line of sight to outcomes. Expectations are well managed because policymakers know what is and is not on offer, including what form the findings will take. 

### Translational bypass

Policymakers are bombarded with information so tend to limit their receptivity to survive, and not all are equipped to understand epidemiological research [7]. Consequently, researchers often struggle to bring even the most relevant research to policymakers’ attention. However, when policymakers are involved as critical thinkers in aspects of the research process, translation is effectively “built in” [9]. Ongoing dialogue also maintains the focus on policy-relevant outcomes, preventing “ivory tower creep” as the project proceeds (e.g. [36, 37]). 

### Efficiency

OOPE maximizes the use of everyone’s time and ensures that each party plays to their strengths: policymakers can critique a tangible plan rather than attempting to articulate one from scratch, and can focus on strategic considerations, leaving the researchers to manage the operational details. 

### Relationship-building

OOPE showcases team flexibility and appreciation of policy realities, as well as research expertise, allowing policymakers to see that these researchers using it are people they can work with. As the literature indicates [26, 38], mutual respect and the development of trust have been vital. Like others, we have found that the social capital generated from one piece of partnered work can drive further collaborations [39]; thus the initial engagement triggers a virtuous circle. We also function as linkage agents in a wider evidence-informed policy network by introducing contacts from academia, the nongovernmental sector and other policy departments [18].

### Mutual learning

Researchers tend to be dismissive about policymakers’ failure to use evidence, but often fail themselves to understand the reasons for this [40]. Our team has learned an enormous amount from working with policymakers which has enhanced our ability to coproduce policy-useful research. Similarly, we have witnessed increasing research sophistication from policy partners during the course of a project. This form of experiential learning-by-doing creates tacit understanding that cannot be matched by journal articles or textbooks [41–43]. Such learning oils the wheels for further collaboration.

### Risk minimization

The non-transactional nature of OOPE avoids problems associated with contractual research—policy agencies are not out of pocket and researchers are not subject to publishing constraints or prevented from conducting research that challenges policy programs or paradigms [39, 44]. Even if nothing comes of initial meetings, the team and work have had exposure in policy circles which can put them on the map for consideration at a later date. 

### Invested policy-makers

In our experience, policymakers who have been involved in shaping our work also champion the use of the research findings within their agencies and beyond. Others attribute this to a sense of ownership of the research [45, 46]. Policymakers have also facilitated introductions to other agencies for related work, enabling new research—policy partnerships.

### Tangible policy impacts

Perhaps most importantly, the approach provides policymakers with access to high-quality evidence to inform policy, and OOPE-informed work has had demonstrable policy impacts, including those illustrated by the case study [31]. 

Other empirical studies of cross-sector participation in research processes show similar effects (e.g. [21, 35, 47]). Further, we believe that OOPE has enabled us to avoid many of the pitfalls that others have encountered, such as repeated meetings with limited impact or outcomes; tokenistic engagement with policymakers resulting in research outputs with poor policy relevance; researchers feeling compromised or politically conflicted by policy relationships; researchers being perceived as arrogant yet also policy-naïve; and finding that the partnership is unsustainable, often due to insufficient institutional support (e.g. [36, 37, 48–50]). The approach acknowledges that evidence is one input of many into policy processes which are often tackling wicked problems where there are diverse stakeholder viewpoints, conflicting values and different assessments of uncertainty and complexity [51]. We aim to contribute empirically reliable, contextually relevant and informative findings, and to support their incorporation in this complex and negotiated process. 

However, there are important limitations. Our ability to respond to policy needs is premised on the epidemiological focus of our research which allows comparatively fast turnarounds, and accurate estimates of what the process and deliverables will entail. Other forms of research, e.g. experimental or qualitative research, would present different challenges. Further, OOPE requires high-quality data and a strong research team. We are a well-established multidisciplinary team with access to large, linked data sets, enough surge capacity to conduct aspects of our work responsively, and specializations that enable us to tackle a wide range of research questions. Our ongoing success in obtaining funding outside of the OOPE process means we are presenting useful information and opportunities to policymakers rather than asking for resources—this frames the encounter far more positively than the “cap-in-hand” approach that policymakers are most used to from researchers but will not always be manageable for other research groups.

OOPE is not suited to all situations. For example, it relies on work being underway or able to commence without specific funding, so is less appropriate for work requiring major up-front investment, such as large-scale infrastructure, trials or cohort studies. It is also not suited to work where the process and findings cannot be shared with government or other stakeholders, or where other more formal coproduction mechanisms are being employed. OOPE often requires locally relevant data, which may be considered less impactful in scientific and publication terms than more investigator-driven work that is at the cutting edge of certain types of scientific knowledge.

Lastly, researchers need to understand and respect the policy process if they are to work effectively within it [13, 52]. We are committed to maximum policy impact, which has led us to invest in educational and interactive forums that increase our policy learning and exposure, including mentoring for junior and mid-career researchers. As our sensitivity to policy needs, constraints and practice norms has increased, we find ourselves better able to facilitate dialogue, anticipate challenges and build shared understanding. But this demands considerable time and resources, and the results are not guaranteed: initial engagement does not always lead to fruitful collaboration, and we have no control over how our work will finally be used by policymakers or practitioners.

## Implications for epidemiological research teams

There are many approaches to research-policy engagement; no single model can work in all circumstances [39], and researchers, policymakers and practitioners must make the most of local opportunities [53]. While many of the characteristics of OOPE are context-specific (developed to meet our team’s circumstances), we believe the processes and principles that underpin it are transferable, and we suggest that other epidemiologists, and potentially other researchers, consider whether this approach might be applied in their contexts. Consequently, we suggest the following:

1. Get in contact with policymakers who work in areas that could be better informed by your research. We have found this to be most effective when engaging across different levels of seniority, particularly with mid-level, more specialized staff.
2. Ask them, “*How can we make our work more useful to you?*”
3. Seek a platform for dialogue and relationship-building that may lead to other shared endeavours.
4. Refine your ability to present your work in an engaging, open way that invites collaborative input.
5. Be prepared to work flexibly when it will not compromise your research interests or integrity, including being clear about the limits of your expertise and capacity.
6. Act opportunistically as well as plan for the long haul; make sure you follow up conscientiously on contacts and commitments.
7. Recognize and use the different forms of policy and research knowledge that inclusive working processes will reveal.

Formal coproduction partnerships (where policymakers and researchers share responsibility for all stages of the research project) are increasingly important mechanisms for aligning research outputs with policy needs, but there is room for other strategies such as OOPE where researchers engage more organically on the basis of improving specific research outputs. Descriptions of such experiences, and of the principles that underpin them, remain rare, so this paper makes a useful contribution to ongoing conversation and research about how to ensure research is relevant and makes a positive difference to policy and practice.

## Data Availability

Not applicable.

## Abbreviations

OOPE: Output-orientated policy engagement
CVD: Cardiovascular disease
RACGP: Royal Australian College of General Practitioners
